# 

**Published:** 1912-12

**Authors:** 


					DECEMBER, 1912.
Vol.
XXX. No. 118,
THE
BRISTOL
medico-chirurgical
NAL
THE BRISTOL MED1CO-CHIRURGICAL SOC1ETT.
Editor: R. SHINGLETON SMITH, M.D., B.Sc.
WITH WHOM ARK ASSOCIATKI)
J. MICHELL CLARKE, M.A., M.D.,
J. LACY FIRTH, M.S., M.D., JAMES SWAIN, M.S., M.D.
P. WATSON-WILLIAMS, M.D., Assistant Editor.
Editorial Sicrttary: J. A. NIXON, B.A., M B.
6a ,
BRISTOL: J. W. ARRO\^Q^ftS^^ 0^ "
London : J. & A. Churchill, 7 Gkkat
Price One Shilling and Sixpence.

				

